# Data-driven analysis of Armeo Spring performance across neurological disorders: implications for personalized upper limb neurorehabilitation

**DOI:** 10.3389/frobt.2026.1773515

**Published:** 2026-02-13

**Authors:** Maria Lui, Desirèe Latella, Luigi Chiricosta, Mauro Botindari, Angelo Quartarone, Mirjam Bonanno, Rocco Salvatore Calabrò

**Affiliations:** IRCCS Centro Neurolesi “Bonino-Pulejo”, Messina, Italy

**Keywords:** data-driven approach, neurorehabilitation, performance metrics, personalized medicine, upper limb robotic rehabilitation

## Abstract

**Background:**

Robotic-assisted therapy (RAT) has emerged as an effective approach to upper limb neurorehabilitation. Among available systems, the Armeo®Spring enables task-oriented, customizable training supported by virtual reality (VR), fostering motivation and neuroplasticity. This retrospective observational study aimed to evaluate longitudinal changes in performance across different VR exercises using Armeo®Spring session data from patients with diverse neurological conditions and to identify tasks exhibiting significant improvement within particular diagnoses, thereby supporting personalized robotic rehabilitation.

**Methods:**

The dataset included adults (≥18) with common neurological disorders who completed ≥20 Armeo®Spring sessions using frequent integrated VR exercises. Performance across the first 20 sessions was analyzed using linear mixed-effects models with fixed effects for session, disease, age, sex, difficulty, and mechanical support, and random patient intercepts and slopes. False discovery rate (FDR) correction was applied to identify disease- and task-specific improvement trajectories.

**Results:**

After sequential filtering, the final cohort included 71 patients (30 with ischemic stroke, 15 with hemorrhagic stroke, 15 with multiple sclerosis, and 11 with Parkinson’s disease) who underwent rehabilitation using five different VR exercises: Balloons, Roll the Ball, Fly High–Elbow, The Goalkeeper, and Pirate Adventure. A significant improvement in *Roll the Ball* scores was detected for MS (slope = +9.41 points/session, FDR = 0.0015), IS (+9.18 points/session, FDR = 0.0001), and HS (+7.28 points/session, FDR = 0.023). In Fly High (Elbow), MS patients demonstrated a significant improvement (+6.84 points/session, FDR <0.001) as for IS patients (+5.00 points/session, FDR <0.001). Task difficulty was consistently correlated with lower scores across all games (FDR <0.05), whereas age and sex were not significant predictors in the adjusted models.

**Conclusion:**

Disease-specific recovery profiles suggest that proximal, multi-joint VR exercises, such as Roll the Ball and Fly High (Elbow), may be particularly effective for patients with multiple sclerosis and ischemic stroke, whereas other exercises show smaller or non-significant improvements. These findings support tailoring VR-based rehabilitation to the patient’s neurological condition, enabling targeted, condition-specific exercise selection and progression, which may enhance the effectiveness and efficiency of upper-limb recovery.

## 1 Introduction

Robotic-assisted therapy (RAT) is a well-established approach for upper limb rehabilitation in neurological and neurodegenerative populations, allowing clinicians to increase the intensity and standardization of therapy. A key strength of RAT is the possibility to personalize treatment in real-time based on a patient’s performance, offering therapy that tailors the levels of difficulties, intensity, required movements, assistance levels, and workspace according to the patient’s progress (Bonanno et al., 2024). This feature not only enhances motor recovery but also improves engagement and motivation, promoting neuroplasticity, a critical factor in the long-term recovery of neurological functions. Indeed, these systems provide multimodal performance feedback through audio and visual stimulation related to task execution and score performance. This aspect, also known as “gamification”, has shown promising benefits in neurorehabilitation, particularly in enhancing patient motivation and enjoyment during training (Adlakha et al., 2020; Tosto-Mancuso et al., 2022). By providing immediate feedback, gamified rehabilitation keeps patients aware of their ongoing performance and therapeutic progress (Huang et al., 2023). Such feedback is typically delivered through multimodal cues, auditory, visual, and textual, that appear instantly after each action, informing patients about task outcomes and overall improvements (Maggio et al., 2019). Moreover, the reward-based scoring mechanisms integrated into many robotic systems, which assign rewards or points based on task completion, can foster self-satisfaction and self-esteem, by reinforcing motivation and promoting emotional engagement, according to previous studies (Maggio et al., 2019; 2023; Hao et al., 2023) Beyond enhancing engagement through multimodal performance feedback and gamified tasks, robotic devices also enable highly standardized and reproducible therapy delivery by controlling task parameters and training dose.

Intensive and repetitive rehabilitation training are essential for regaining motor functions, which can be challenging to achieve with traditional therapy (Calabrò et al., 2021). Several studies, including systematic reviews, have shown that RAT not only improves activities of daily living but also enhances muscle strength and function in the affected arm (Mehrholz et al., 2020; Straudi et al., 2022). Two recent large-scale studies further corroborate these findings, showing that RAT is at least as effective as conventional therapy in improving motor outcomes. 16. The most recent meta-analysis suggests that robotics can improve upper limb motor function and muscle strength (Mehrholz et al., 2020), and, when compared to a similar amount of conventional therapy, no significant differences in terms of motor recovery are detected (Aprile et al., 2020).

Nevertheless, motor recovery is often evaluated through clinical scales that are dependent on clinician observation. From a therapeutic perspective, robotic platforms enable high-dose, task-oriented practice with standardized task parameters and the possibility to adjust difficulty and assistance during training. In addition, the same devices can continuously record objective, quantitative performance data (e.g., scores and kinematic proxies), which may complement clinical scales but remain less explored in the current literature (Bonanno and Calabrò, 2023).

Among the different robotic systems available for upper-limb rehabilitation, the Armeo®Spring (Hocoma AG, Switzerland) is a gravity-supporting exoskeleton capable of providing customizable levels of arm weight compensation. The device is integrated with a virtual reality (VR) screen through which patients can interact and perform goal-oriented tasks by moving their arm. Thanks to its mechanical structure, the Armeo®Spring allows therapists to train the entire movement chain from shoulder to hand, while selectively blocking joints to focus on specific movements tailored to each patient’s needs. Each proposed exercise is customable in terms of level of difficulty, transforming repetitive therapy into interactive and personalised tasks that encourage higher intensity and sustained participation over the training sessions (Gijbels et al., 2011; Perry et al., 2017; Olczak et al., 2022). It is worth noting that Armeo®Spring has demonstrated benefits across different neurological conditions, including stroke, multiple sclerosis, and Parkinson’s disease (Gijbels et al., 2011; Taveggia et al., 2016; Raciti et al., 2022). However, these disorders are characterized by distinct motor phenotypes, post-stroke impairment often involves paresis and reduced active workspace (with hemorrhagic stroke frequently showing greater initial severity and slower early recovery) (Saes et al., 2022), MS may be influenced by fatigue and day-to-day performance fluctuations despite preserved learning capacity (Valè et al., 2020; Ingram et al., 2022), and PD is typically dominated by bradykinesia, impaired timing, and coordination deficits (Capato et al., 2023). As a result, even when patients engage in largely similar Armeo Spring games, the same VR task may not be equally informative or equally responsive to change across conditions. Therefore, rather than assuming comparable suitability or efficacy of Armeo®Spring across disorders, the present study leveraged routinely collected device logs to test whether longitudinal, session-by-session performance trajectories on the same exercises are task- and disease-specific (Argunsah et al., 2026), after accounting for key training-related covariates (e.g., difficulty, mechanical support, and therapy dose). Beyond their therapeutic role, modern exoskeletons and rehabilitation robots are also being recognized as measurement platforms, because embedded sensors and device logs can provide objective, reproducible performance indicators that may complement traditional clinician-rated scales and support more data-driven monitoring. At the same time, recent advances in AI-enabled rehabilitation robotics emphasize the potential of continuous data capture and automated progress tracking to inform personalization and progression of therapy, while also underscoring the need for clinically grounded interpretation and validation of device-derived metrics (Abbas et al., 2025). Neurological populations further add complexity due to intrinsic heterogeneity; in Parkinson’s disease, for example, data-driven phenotyping approaches using objective biomarkers have shown that distinct subtypes may exhibit different longitudinal rehabilitation responses, highlighting the value of longitudinal quantitative profiling beyond conventional outcomes alone (Wang et al., 2026). Within this context, an important open question is whether routinely collected device logs can reliably capture session-by-session performance trajectories across tasks and diagnoses in real-world clinical practice.

Most Armeo®Spring evidence is based on single-diagnosis pre–post studies and/or a limited set of device-derived parameters, with psychometric work indicating that learning/familiarization can affect device metrics (Gijbels et al., 2011; Taveggia et al., 2016). However, routine-log studies that model session-by-session trajectories across multiple games and compare patterns across different neurological disorders within a unified framework remain scarce.

This retrospective observational study aimed to analyze longitudinal changes in performance across different VR exercises on Armeo®Spring device over 20 consecutive rehabilitation sessions in patients with ischemic stroke, hemorrhagic stroke, multiple sclerosis or Parkinson’s disease. Specifically, we sought to identify, within each neurological condition, which VR-based tasks show significant improvement over time. We hypothesized that patients would show progress over repeated sessions, but that patterns of improvement would vary based on the neurological conditions and motor components emphasized by each task. The purpose of this analysis is to support the development of personalized rehabilitation strategies by the characterization of disease- and task-specific outcomes, thereby informing a more targeted selection and progression of VR exercises in clinical practice.

## 2 Materials and methods

### 2.1 Study design and data source

This retrospective study analyzed data from neurological patients who underwent robotic rehabilitation with the Armeo®Spring device at the Robotic Neurorehabilitation Unit of IRCCS Centro Neurolesi “Bonino Pulejo”, Messina, Italy, between 2017 and 2025. Data were retrieved from medical records and Armeo®Spring device logs, including demographic, clinical, and exercise-related variables. Patients were eligible if they were 18 years or older and had completed at least 20 Armeo®Spring sessions, each lasting approximately 60 min, in order to ensure sufficient exposure for analysis. This cut-off was defined as a priori, as upper limb robotic rehabilitation protocols frequently include approximately 3–5 weeks of training (about 15–20 sessions), which is usually sufficient to detect changes in task-specific performance. In our inpatient setting, sessions are routinely delivered as part of standard care and last 60 min; therefore, 20 sessions correspond to around 20 h of structured, task-oriented training, consistent with guideline-level recommendations (Winstein et al., 2016; Calabrò et al., 2021; Alt Murphy et al., 2025) emphasizing sufficient therapy intensity/dose to support upper-limb recovery. Patients with fewer than 20 sessions were excluded to ensure that individual recovery profiles were based on a comparable minimum exposure to the intervention. According to institutional policies and applicable regulations, this study was exempt from ethics committee approval, as it involved retrospective analysis of fully anonymized data collected during routine clinical care. Patients were informed at admission that their anonymized clinical data could be used for research purposes. Moreover, given its retrospective nature, no additional intervention or assessment was performed for the purpose of this study.

### 2.2 Study population and filtering steps

Our retrospective analysis included 173 patients (108 males, 65 females) who underwent Armeo®Spring–based upper limb rehabilitation between 2017 and 2025, with ages at treatment ranging from 19 to 84 years. The treated limb was the left arm in 80 patients, the right arm in 90 patients, and both arms in 3 patients. The study population included individuals diagnosed with the following conditions:

- Ischemic Stroke (61 patients)
- Multiple Sclerosis (34 patients)
- Hemorrhagic Stroke (24 patients)
- Parkinson’s Disease (20 patients)
- Traumatic Brain Injury (15 patients)
- Brain tumor (6 patients)
- Spinal Cord Injury (4 patients)
- Guillain–Barré Syndrome (4 patients)
- Amyotrophic Lateral Sclerosis (2 patients)
- Familial Spastic Paraparesis (1 patient)
- Charcot–Marie–Tooth Disease (1 patient)
- Muscular dystrophy (1 patient)

Patients underwent a variable number of Armeo®Spring rehabilitation sessions, ranging from 2 to 267. Data preprocessing was performed in two sequential filtering steps. First, to reduce bias related to underrepresented conditions, only patients diagnosed with the most prevalent neurological diseases in the cohort were retained: Parkinson’s Disease (PD), Multiple Sclerosis (MS), Hemorrhagic Stroke (HS), and Ischemic Stroke (IS). The original dataset of 173 patients was therefore reduced to 139 individuals who had performed at least one Armeo®Spring session. Then, to ensure task consistency across subjects, we examined the frequency of virtual games usage across the ones available in the Armeo®Spring system thereby highlighting these patients most frequently engaged in five VR tasks: Balloons (104 patients), Pirate Adventure (86), Goalkeeper (88), Fly High (Elbow) (90) and Roll the Ball (87). Subsequently, a second filter was applied to ensure a comparable number of sessions among patients included in the final dataset. We therefore retained only patients who had completed at least 20 Armeo®Spring therapy sessions involving these five games. After applying these inclusion criteria, the final study cohort therefore comprised 71 patients, who formed the population for all subsequent analyses. This final dataset included 30 patients with ischemic stroke (IS), 15 with multiple sclerosis (MS), 15 with hemorrhagic stroke (HS) and 11 with Parkinson’s disease (PD). Within this cohort, the number of patients who completed at least 20 sessions for each game was as follows: Balloons (n = 33), Pirate Adventure (n = 28), Goalkeeper (n = 36), Fly High (Elbow) (n = 36) and Roll the Ball (n = 33). The progressive filtering steps applied to the Armeo®Spring dataset are summarized in Supplementary Figure S1.

**FIGURE 1:**
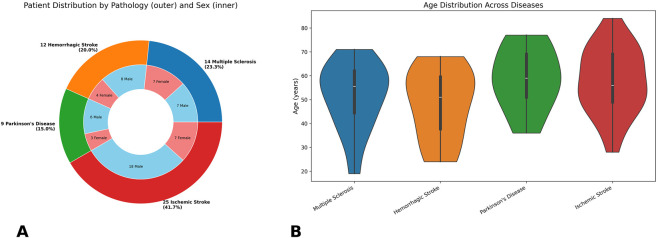
Clinical and demographic description of the study samples. **(A)** Distribution of patients by pathology and sex represented on a polar bar chart: The outer ring illustrates the proportion of patients for each disease. The inner ring subdivides each pathology segment by sex, with blue representing males and red representing females. This visualization highlights both the relative prevalence of each condition and the gender balance within each disease group. **(B)** Age distribution of patients across diseases under investigation. Each violin shows the kernel density estimate of ages within each pathology group, with an internal boxplot indicating the interquartile range and median age thereby highlighting both the central tendency and variability of patient ages within each disease group.

The patient distribution by pathology and sex was reported in (Figure 1A) together with the age distribution across diseases reported in (Figure 1B).

### 2.3 Description of the rehabilitation program

Upper-limb robotic rehabilitation was performed using the Armeo®Spring (Hocoma AG, Switzerland), a gravity-supporting exoskeleton integrated with a virtual reality (VR) environment. The device allows intensive, task-oriented motor training of the shoulder, elbow, and wrist through interactive game-like exercises providing augmented performance feedback. The mechanical support can be adjusted according to the patient’s motor capabilities, enabling training across different levels of impairment severity. Performance data (scores) obtained from the Armeo®Spring’s Augmented Performance Feedback and VR exercises were extracted and analyzed to assess within-disease improvements over time and to compare the relative effectiveness of different exercises among disease groups. The data report generally records difficulty, time of therapy, and arm/forearm weight support. In particular, task difficulty is defined as a three-level ordinal setting (easy, medium, hard). Higher difficulty levels were associated with more distractors, smaller and more numerous targets, and faster scene dynamics, thereby increasing the demands on accuracy, visuomotor coordination and reaction time. Arm and forearm weight support are set on a nine-level spring scale (A–I), where A indicates minimal and maximal weight offloading, with B–H providing intermediate support levels. Finally, for each patient and session, therapy minutes denoted the cumulative duration of Armeo®Spring training (in minutes) delivered up to and including that session.

The analysis focused on five Armeo®Spring exercises: Balloons, Fly High Elbow, The Goalkeeper, Pirate Adventure, and Roll the Ball, which represent the most frequently prescribed VR gamified tasks (see Table 1). These exercises ultimately target a range of upper-limb motor functions, including shoulder and elbow control, coordination, and strength.

**TABLE 1:** Description of the performed exercises.

Exercise	Target movements	Description	Trained functions
Balloons	Flexion-extension of the shoulder; abduction-adduction of the shoulder and circumduction	The subject has to move his/her shoulder in order to take all the ballons in different directions, also avoiding bombs	Multidirectional proximal upper limb control and spatial exploration during reaching
Roll the ball	Flexion-extension of the shoulder; abduction-adduction of the shoulder and flexion/extension of the elbow	The subject needs to move his/her shoulder and elbow in order to collect every coin in the scene, avoiding bombs	Coordinated shoulder–elbow reaching with trajectory accuracy for goal-directed movements
Pirate adventure	Flexion-extension of the shoulder; abduction-adduction of the shoulder and flexion/extension of the elbow	The subject needs to move his/her shoulder and elbow in order to avoid knives from pirates	Rapid, selective upper limb responses to moving stimuli, enhancing visuomotor coordination and reaction capacity
Fly high (elbow)	Flexion/extension of the elbow	The subject moves his/her elbow in order to catch each coin on the screen, avoiding birds	Selective elbow control and graded reaching towards/away from the body
Goalkeeper	Prono-supination of the forearm	The subject has to move his\her forearm in order to catch soccer balls of different sizes, avoiding other objects, such as shoes	Selective forearm pronation–supination for distal orientation control in visuomotor tasks

In addition to robotic training, patients received concomitant conventional rehabilitation as part of routine inpatient care during the same weeks. Conventional therapy was individualized according to clinical needs and typically included passive and active-assisted mobilization, stretching, and range-of-motion/mobility exercises, complemented by task-oriented functional practice when appropriate, in line with the multidisciplinary pathway adopted at our Institute (Manuli et al., 2020; Maggio et al., 2024; Maggio et al., 2024; Manuli et al., 2020). The relative timing of conventional therapy with respect to the Armeo session (before/after) was not standardized and depended on daily scheduling and patient tolerance.

### 2.4 Data parsing and analysis

Data collected from Armeo®Spring were iteratively parsed with an in-house coded Python script (Supplementary Material–Data Sheet 1) for the reconciliation of sparse data from each patient into structured format to read, process and use for following statistical analysis. Date of birth and date of physiotherapy sessions were used to compute the age for each patient at the time of rehabilitation, all metadata were either transformed into categorical variables (as for exercise difficulty level, patient sex, and arm/forearm weight support) or left as quantitative (such scores). Data parsing, statistical analyses, and graphical representations were performed using the pandas (version 2.2.2; (McKinney, 2010), NumPy (version 1.26.4; (Harris et al., 2020), Matplotlib (version 3.9.1; (Hunter, 2007), Seaborn (version 0.13.2; (Waskom, 2021), and statsmodels (version 0.14.1 (Seabold and Perktold, 2010); libraries in Python (version 3.9.12; (Python 3 Reference Manual, 2025).

For each Armeo®Spring game under investigation and for each disease type included in our final dataset, we examined the relationships among the quantitative variables though pairwise Pearson correlation coefficients computed with Python. Numeric features were used to measure the strength and direction of linear associations between continuous variables resulting in correlation coefficients ranging from −1 (perfect negative linear relationship) to +1 (perfect positive linear relationship) and plotted as heatmap matrices using Seaborn package. The magnitude of correlation coefficients was interpreted according to standard thresholds described by Mukaka (Mukaka, 2012) with values from 0.00 to 0.30 considered negligible, 0.30 to 0.50 considered low, 0.50 to 0.70 considered moderate to strong, 0.70 to 0.90 considered strong, and 0.90 to 1.00 considered very strong. These thresholds were applied consistently across all analyses to describe the strength of associations between rehabilitation parameters and patient performance metrics.

In order to evaluate the progression of patients during ArmeoSpring rehabilitation and the influence of key covariates, for each selected game, patient scores across 20 rehabilitation sessions were analyzed using a linear mixed-effects modeling (LMM) approach (Pinheiro, 2014) implemented in python with the statsmodel library. Mixed models are particularly suitable for studies with repeated measurements of the same variable from the same participant over time (e.g., longitudinal studies). Specifically, our LMM integrated both fixed effects and random effects in the analysis, and the dependent variable was the game performance score. The fixed effects included the session number, sex, age and disease type, difficulty of the exercise, forearm and arm weight support levels. Specifically, disease type was used as an interaction term with the session number to capture disease-specific trends in improvement. Fixed effects were specified to estimate population-average associations between these covariates and performance, consistent with standard practice in longitudinal designs where experimental or demographic factors are controlled by design (West et al., 2014; Introduction to Linear Mixed Models, 2025).In linear mixed-effects models, random effects represent subject-specific deviations from the population-average relationship and are modeled as realizations from an underlying population distribution, allowing the partitioning of variability into within- and between-subject components (West et al., 2014). To account for repeated measures and inter-individual variability in rehabilitation trajectories, each patient was therefore modeled with a random intercept and a random slope for session number. This random-effects structure allowed individual differences in both baseline performance and rate of improvement over time and is appropriate when observations are nested within higher-level units, such as rehabilitation sessions within patients (West et al., 2014).

For each disease category, the intercept represented the estimated baseline score at the first session, while the slope quantified the mean change in performance per session, reflecting the rate of improvement. To ensure consistent terminology for describing the magnitude of improvement, daily slopes (points per session) were classified as negligible for values below 2 points/session, low for values between 2 and 5 points/session, moderate for values between 5 and 8 points/session, and strong for values greater than 8 points/session. Random intercepts and slopes were assumed to follow a joint normal distribution and were estimated jointly with fixed effects and variance components. The linear mixed-effects model can therefore be summarized with the following mathematical formulas: $score_{ij}=\sum_{k=1}^{k}\beta_{k}X_{ijk}+b_{0i\;}+b_{1i}Day_{ij}+\varepsilon_{ij}$ Where:

-
$score_{ij}$ resents the observed performance score for patient *i* during rehabilitation session *j*.
-
$X_{ijk}$ denotes the value of the $k^{th}$ fixed-effect predictor (Pathology, the interaction Day × Pathology, Difficulty, Age, Sex, Forearm Support, and Arm Support) for patient *i* during rehabilitation session *j*.
-
$\beta_{k}$ represents the corresponding fixed-effect coefficient, quantifying the average influence of predictor $X_{ijk}$ on the score across all patients.
-
$b_{0i}$ is the random intercept for patient *i,* accounting for individual baseline differences in performance, and representing a patient-specific deviation from the population-average intercept (indicates how much patient *i* starts above or below the average baseline performance).
-
$b_{1i}$ is the random slope for Day for patient *i,* allowing each patient to have its own rate of chance across rehabilitation sessions, and represents how much faster or slower patient *i* improves (or declines) per training session compared to the average learning rate.
-
$\varepsilon_{ij}$ is the residual error term, representing within-patient unexplained variability.

Two complementary statistical assessments were performed, both using the Wald test (Luke, 2017). The first one aimed to verify whether the disease-specific improvement for a specific game was significant or not by estimating the rate of change in performance for each disease. Specifically, meaningful improvements over the sessions were characterized by slope coefficients significantly different from zero. The second Wald test was performed for the estimation of covariate effects. Fixed effects for age, sex, game difficulty, and arm support were therefore evaluated to determine whether these factors independently influenced performance, regardless of disease or session number. For each fixed effect variable, we computed its effect on score, together with its associated p-values, to eventually quantify their contribution to the improvement. To mitigate the risk of type I error due to multiple testing, False Discovery Rate (FDR) correction using the Benjamini–Hochberg procedure was applied separately to p-values for slopes, intercepts, and covariates. Statistical significance was defined as an FDR-adjusted p < 0.05.

## 3 Results

### 3.1 Effect of age, sex and exercise difficulty on improvement

**FIGURE 2:**
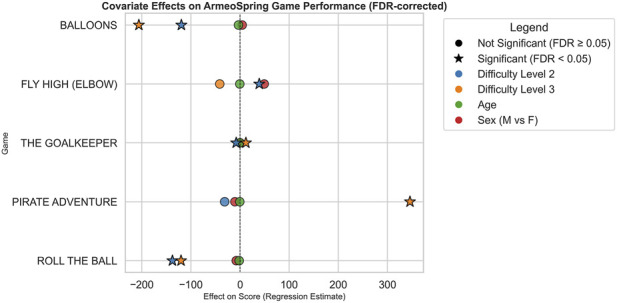
Covariate effects on ArmeoSpring game performance after FDR correction. Each point represents the estimated regression effect of a specific covariate on performance scores across the five ArmeoSpring games under investigation. Covariates include Difficulty Level, Age, and Sex and are differently represented using a different color-code. Stars denote statistically significant effects after false discovery rate (FDR) correction (FDR <0.05), while circles indicate non-significant results (FDR ≥0.05). Positive values on the x-axis correspond to covariates associated with higher performance scores, whereas negative values indicate covariates associated with lower performances. The dashed vertical line at zero marks the null effect threshold.

Covariate effect across games is represented in Figure 2 highlighting which covariates significantly influenced scores, the direction and magnitude of effects, and how patterns differ across games.

Difficulty levels showed strong and statistically significant effects, consistently associated with lower scores. This pattern aligns with expectations, confirming that task difficulty is a major determinant of performance and highlights the importance of adjusting difficulty to individual patient abilities during rehabilitation. In contrast, the effects of sex and age were non-significant across games, suggesting that, within this patient cohort, performance improvements during Armeo®Spring rehabilitation were not systematically influenced by gender or age.

Although exploratory correlation analyses suggested some age-related patterns within specific small subgroups (e.g., PD), age did not emerge as a consistent independent predictor of performance in the mixed-effects models once all covariates and repeated measures were considered. These subgroup correlations should therefore be interpreted with caution.

### 3.2 Disease-specific performance and improvement trends

The linear mixed-effects models estimated baseline performance (intercepts) and daily improvement rates (slopes per training session) for each disease across the five selected Armeo®Spring games.

**FIGURE 3:**
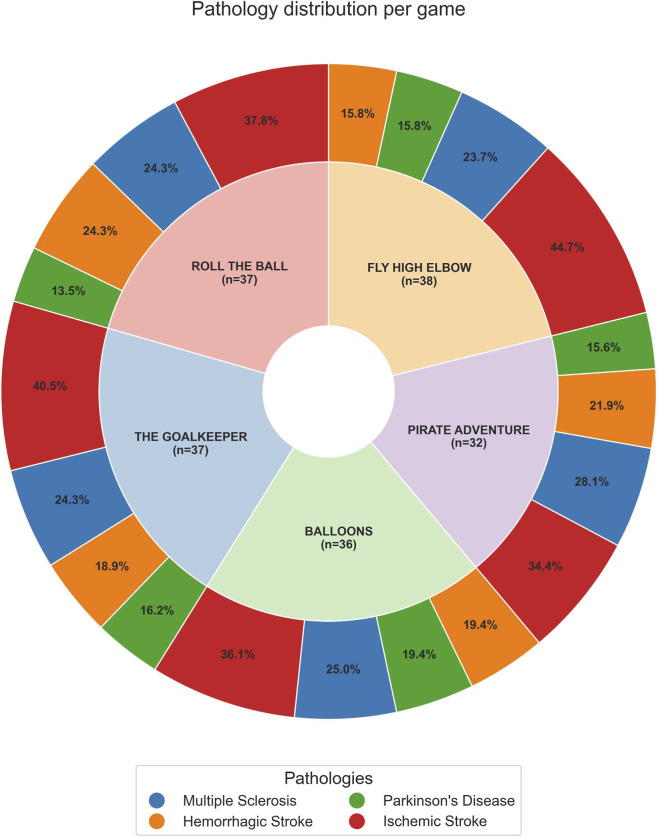
Nested pie chart showing the distribution of patient pathologies across different games. The inner ring represents each VR game, with labels indicating the game name and total number of patients. The outer ringrepresents the distribution of pathologies within each game, with percentages showing the proportion of patients with each pathology relative to that game. The legend at the bottom identifies the colors corresponding to each pathology.

**FIGURE 4:**
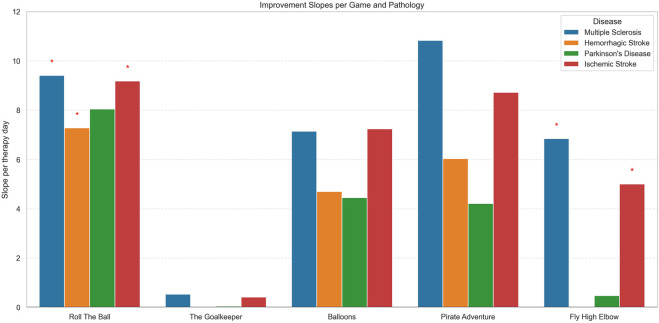
Bar plot showing the mean improvement slopes per therapy day for each game, stratified by pathology. Each colored bar represents a disease: Multiple Sclerosis, Hemorrhagic Stroke, Parkinson’s Disease, or Ischemic Stroke. Red asterisks above bars indicate slopes that are statistically significant after false discovery rate (FDR) correction. The plot allows comparison of improvement rates across different games and pathologies, highlighting which patient groups show significant progress.

Wald tests with false discovery rate (FDR) correction using the Benjamini–Hochberg procedure were applied to assess statistical significance, which was defined as an FDR-adjusted p-value <0.05. For each rehabilitation game, we examined both the distribution of patients across neurological diseases (summarized in Figure 3) and the corresponding daily improvements in performance (summarized in Figure 4). The specific distribution of neurological diseases and the daily improvement in performance for each disease are reported for each VR rehabilitation game in the supplementary figures (Supplementary Figures S2–S6).

Figure 3 shows, for each VR game, the proportion of patients for each disease, providing context for the relative representation of MS, IS, HS, and PD in our sample.

Figure 4 presents the daily improvement in scores (slope per therapy day) for each VR game and for each disease, highlighting differences in responsiveness to the specific game. Moreover, we assessed whether the improvement was statistically significant, accounting for inter-patient variability and multiple testing (FDR correction), and statistically significant results were highlighted with red star symbols.

Across most games, positive slopes were observed, indicating improvement in game performance with repeated sessions. However, the magnitude and significance of improvement varied by disease and game type.

For the Roll the Ball exercise, patients with MS, IS, and HS patients achieved the largest daily improvements, with slope of approximately 9.41 (p < 0.001, FDR = 0.0015), 9.18 (p < 0.001, FDR = 0.0001) and 7.28 (p = 0.006, FDR = 0.023) points per rehabilitation session, respectively, and all significant after FDR correction. PD patients showed moderate improvements (8.05 points/session) but were not statistically significant.

**FIGURE 5:**
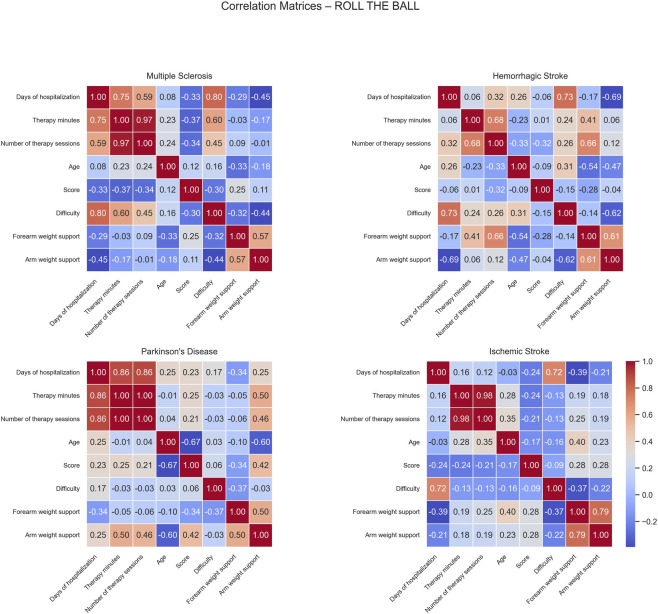
Correlation matrices of performance and clinical variables by pathology for the Roll The Ball exercise. Each subplot displays the pairwise Pearson correlation coefficients among numerical performance and clinical variables within a specific disease group. Color intensity represents the strength and direction of the correlation, with blue indicating positive and red indicating negative relationships. Only variables with sufficient variability are included.

For MS patients, strong positive correlations were observed between difficulty and both days of hospitalization (0.80) and therapy minutes (0.60), indicating that longer hospital stays and therapy duration were associated with more intensive and challenging therapy programs. No strong negative correlations were observed (Figure 5).

In HS patients, positive correlations were observed between days of hospitalization and difficulty (0.73) and between the number of sessions and forearm weight support (0.66), indicating that patients with longer hospital stays tended to face higher task demands and that patients completing more sessions required greater forearm support. Conversely, negative correlations were found between days of hospitalization and arm weight support (−0.69), and between difficulty and arm weight support (−0.62), and between age and forearm weight support (−0.54). This suggests that patients with longer hospital stays or higher task difficulty required less arm support, while older patients required less forearm support, possibly reflecting improved recovery or adaptive therapy strategies.

For PD patients, negative correlations were noted between age and both score (−0.67) and arm weight support (−0.60), suggesting that older patients achieved lower performance and received less mechanical support.

In IS patients, days of hospitalization showed a strong positive correlation with difficulty (0.72) while no significant negative correlations were observed.

In The Goalkeeper exercise, small improvement was observed primarily in MS and IS patients with a slope characterized by 0.5 and 0.4 points/rehabilitation session but not significant after FDR correction. Whereas HS and PD patients exhibited non-significant daily gains in slope.

**FIGURE 6:**
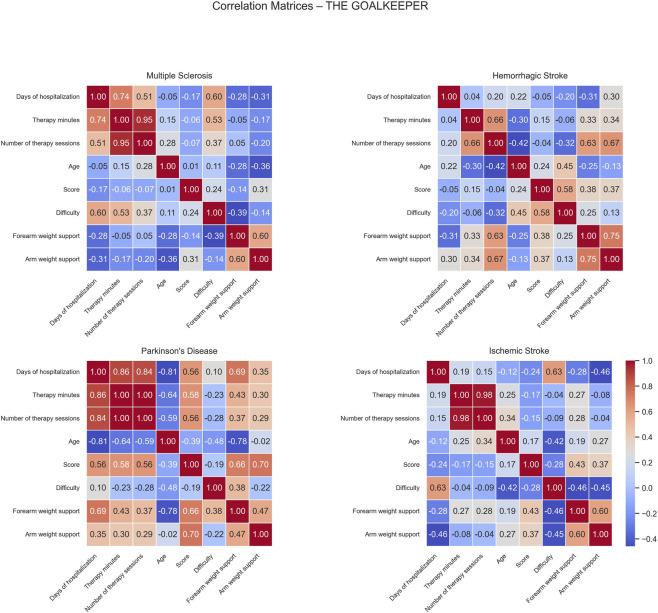
Correlation matrices of performance and clinical variables by pathology for the Goalkeeper exercise. Each subplot displays the pairwise Pearson correlation coefficients among numerical performance and clinical variables within a specific disease group. Color intensity represents the strength and direction of the correlation, with blue indicating positive and red indicating negative relationships. Only variables with sufficient variability are included.

Correlation matrices computed for the Goalkeeper game show different patterns of positive and negative correlations across the patient groups (Figure 6).

For MS patients, moderate positive correlations were observed between difficulty and days of hospitalization (0.60) and therapy minutes (0.53) while no significant negative correlations were found in this group.

In HS patients, positive correlations were identified between number of sessions and both forearm (0.63) and arm weight support (0.67). Additionally, score correlated positively with difficulty (0.58), indicating that patients performing at higher difficulty levels tended to achieve better scores. No strong negative correlations were observed, suggesting generally aligned relationships between therapy intensity, mechanical support, and task performance.

For PD patients, moderate positive correlations emerged between days of hospitalization and both score (0.56) and forearm weight support (0.69). Similarly, score showed positive associations with both forearm (0.66) and arm weight support (0.70), indicating that better-performing patients were characterized by longer hospital stays and received greater mechanical assistance. In contrast, negative correlations were observed between age and days of hospitalization (−0.81), therapy minutes (−0.64), number of sessions (−0.59), and forearm weight support (−0.78), suggesting that older patients generally had shorter, less intensive rehabilitation and received lower mechanical support.

In IS patients, days of hospitalization correlated positively with difficulty (0.63), meaning patients with longer rehabilitation periods might have progressed enough to be engaged with more challenging exercises. No significant negative correlations were found in this group.

For the Balloons exercise, although baseline scores were high across all diseases (Mean ≈320.34) and significantly differs among different diseases (p < 0.001, FDR = 0.00093), the day-to-day improvements were generally small and not statistically significant, indicating limited gains during the first 20 sessions for all patient groups.

**FIGURE 7:**
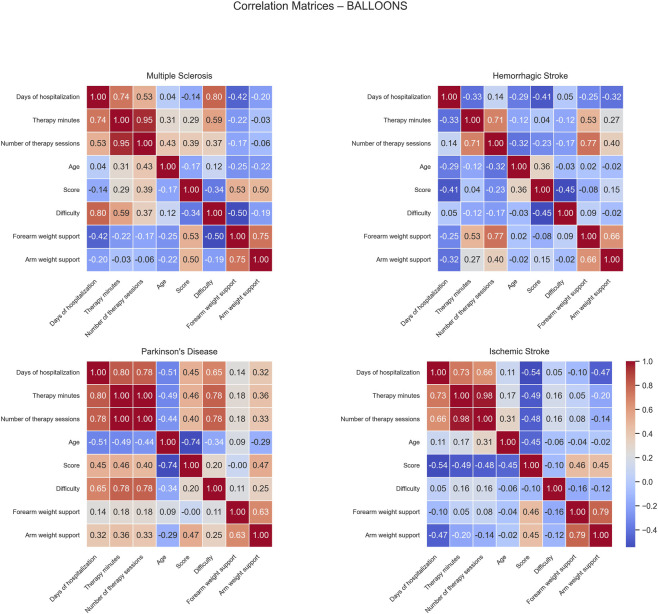
Correlation matrices of performance and clinical variables by pathology for the Balloons exercise. Each subplot displays the pairwise Pearson correlation coefficients among numerical performance and clinical variables within a specific disease group. Color intensity represents the strength and direction of the correlation, with blue indicating positive and red indicating negative relationships. Only variables with sufficient variability are included.

Correlation matrices computed for the Balloons game show distinct patterns of positive and negative correlations across the different patient groups (Figure 7). For MS patients, positive correlations were observed between difficulty and both days of hospitalization (0.80) and therapy minutes (0.59), suggesting that longer hospital stays were associated with more intensive and challenging therapy programs. The score correlated positively with forearm weight support (0.53), indicating mechanical assistance had a beneficial effect on performance overall facilitating the effective participation and success of rehabilitation.

In HS patients, positive correlations were found between forearm weight support and both therapy minutes (0.53) and number of therapy sessions (0.77), suggesting that patients receiving longer or more frequent therapy tended to require greater mechanical assistance. No significant negative correlations were detected.

For PD patients, positive correlations were identified among days of hospitalization, therapy minutes, number of therapy sessions, and difficulty (ranging from 0.65 to 1.00), indicating that therapy duration, frequency, and task challenge were closely linked. In contrast, age showed negative correlations with days of hospitalization (−0.51) and score (−0.74), suggesting that older patients tended to have shorter hospital stays and lower performance outcomes.

In IS patients, a negative moderate correlation was observed between days of hospitalization and score (−0.54), suggesting that longer hospital stays were linked to lower performance levels, possibly reflecting greater initial impairment.

In the Pirate Adventure game, the MS group showed uncorrected evidence of improvement (p = 0.046), but this effect did not remain significant after FDR correction (p = 0.10) and should therefore be interpreted as exploratory. No significant changes were observed for ischemic or hemorrhagic stroke, or for PD.

**FIGURE 8:**
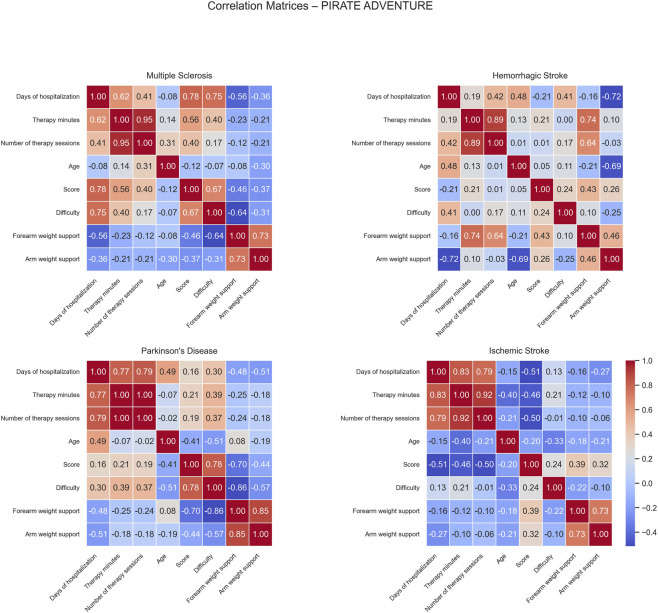
Correlation matrices of performance and clinical variables by pathology for the Pirate Adventure exercise. Each subplot displays the pairwise Pearson correlation coefficients among numerical performance and clinical variables within a specific disease group. Color intensity represents the strength and direction of the correlation, with blue indicating positive and red indicating negative relationships. Only variables with sufficient variability are included.S.

Correlation matrices computed for the Pirate Adventure game show distinct patterns of positive and negative associations across the different patient groups (Figure 8).

For MS patients, positive correlations were observed between days of hospitalization and both score (0.78), and difficulty (0.75), suggesting that longer hospital stays were associated with higher performance and more challenging gameplay. This evidence is also reflected by the positive correlation between therapy minutes and score (0.56), indicating that patients who received more therapy tended to perform better. Score and difficulty were positively correlated (0.67), reflecting that better-performing patients tended to play at higher difficulty levels. Conversely, negative correlations were found between days of hospitalization and forearm weight support (−0.56) and between difficulty and forearm weight support (−0.64), indicating that longer stays and higher task difficulty were associated with reduced forearm support, possibly reflecting improved motor ability.

In HS patients, positive correlations emerged between forearm weight support and both therapy minutes (0.74) and the number of therapy sessions (0.64). These associations suggest that longer rehabilitation therapy exposure was linked with increased mechanical assistance. On the other hand, days of hospitalization correlated negatively with arm weight support (−0.72), and age showed a negative correlation with arm weight support (−0.69), implying that older or longer-stay patients tended to require less arm mechanical support, potentially reflecting progress over the rehabilitation period.

For PD patients, strong positive correlations were observed between score and difficulty (0.78), indicating that higher task difficulty was associated with higher performance scores. Moreover, several negative correlations were identified: days of hospitalization and arm weight support (−0.51), suggesting that patients with longer hospital stays required additional mechanical assistance during training. A moderate negative correlation was also observed between age and difficulty (−0.51), suggesting older patients to generally undergone rehabilitation exercises at easier difficulty levels. Forearm weight support negatively correlated with both score (−0.70), and difficulty (−0.86), suggesting that patients with higher motor impairment and thereby requiring greater mechanical assistance tended to be engaged in less challenging tasks and were overall characterized by lower performances. This evidence was also reinforced by the negative correlation between difficulty and arm weight support (−0.57).

In IS patients, negative correlations were observed between score and both days of hospitalization (−0.51) and number of therapy sessions (−0.50), suggesting that longer or more frequent therapy was associated with lower game performance, possibly reflecting that more severely affected patients required extended rehabilitation.

Finally, for the Fly High Elbow exercise, MS and IS patients demonstrated significant improvement. MS patients showed a trend toward improvement (slope = +6.84 points per rehabilitation session, p < 0.001, FDR <0.001), together with the group of IS patients (slope = +5.00, p < 0.001, FDR <0.001). On the contrary, PD and HS groups did not show significant changes in performance (p > 0.75) in this game under the same training conditions. Notably, HS patients showed no significant change in performance across sessions but were characterized by an overall higher baseline intercept (intercept = 161.2, p = 0.0062, FDR = 0.025), suggesting that these patients started at a higher score level.

**FIGURE 9:**
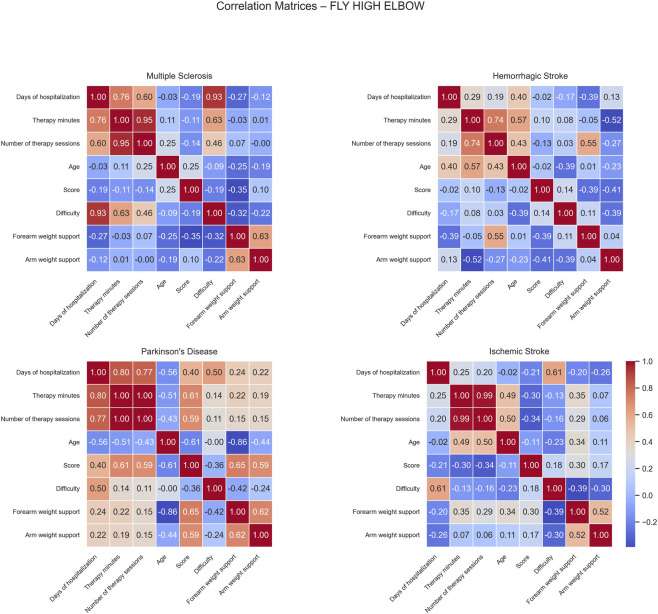
Correlation matrices of performance and clinical variables by pathology for the Fly High Elbow exercise. Each subplot displays the pairwise Pearson correlation coefficients among numerical performance and clinical variables within a specific disease group. Color intensity represents the strength and direction of the correlation, with blue indicating positive and red indicating negative relationships. Only variables with sufficient variability are included.

Correlation matrices computed for the Fly High Elbow game revealed distinct patterns of positive and negative associations across the different patient groups (Figure 9).

For MS patients, positive correlations were observed between difficulty and both days of hospitalization and difficulty (0.93) and therapy minutes (0.63), indicating that longer hospital stays were associated with increased task complexity. No significant negative correlations were found in this group.

In HS patients, positive correlations were identified between therapy minutes and age (0.57), and between the number of therapy sessions and forearm weight support (0.55). These findings indicate that older patients tended to receive more therapy and higher levels of forearm assistance. However, a moderate negative correlation was found between therapy minutes and arm weight support (−0.52), suggesting that increased therapy time was associated with reduced mechanical assistance, possibly due to patient improvement or adaptation during rehabilitation.

For PD patients, moderate positive correlations were observed among score and both therapy minutes (0.61), and number of sessions (0.59). These relationships indicate that greater therapy exposure was associated with better game performance. Score also correlated positively with both forearm (0.65) and arm weight support (0.59), implying that balanced mechanical support may enhance patient engagement and performance. Conversely, age showed several negative correlations: with days of hospitalization (−0.56), therapy minutes (−0.51), score (−0.61), and forearm weight support (−0.86); this suggests that older patients participated less in therapy, performed worse, and required less mechanical support.

In IS patients, moderate positive correlations were observed between days of hospitalization and difficulty (0.61) and number of sessions and age (0.50). These findings suggest that longer therapy periods were associated with higher task difficulty, older patients completed a greater number of sessions, possibly reflecting the need for extended rehabilitation duration or slower recovery profiles in this subgroup. No strong negative correlations were detected in this group.

## 4 Discussion

In this retrospective study, Armeo®Spring game-derived performance scores demonstrated disease- and task-specific recovery profiles across the first 20 rehabilitation sessions. Overall, performance tended to improve repeated sessions in most games; however, improvement patterns differed across neurological conditions and task types. Patients with MS and IS showed the most consistent improvements across the selected VR exercises, with the largest and most robust gains observed in Roll the Ball and Fly High (Elbow). These tasks primarily engage in proximal shoulder and elbow control and require continuous trajectory tracking, which are motor components known to respond well to intensive, task-oriented upper limb training in these populations. In contrast, patients with HS and PD exhibited slower or non-significant improvements under the same VR exercises. The recovery profiles in MS and IS are consistent with evidence that both groups can benefit from structured, repetitive upper limb rehabilitation, often with preserved cognitive resources and endurance that facilitate active participation (French et al., 2016). By comparison, the slower or absent changes observed in HS and PD in our cohort are likely influenced by greater clinical heterogeneity and by the predominant motor impairments in these conditions and should not be interpreted as evidence of a lack of responsiveness to rehabilitation. In HS, upper limb recovery is often characterized by more severe initial motor impairment and lower activity capacity compared to IS. However, Persson et al. (Persson et al., 2016) reported that, despite worse baseline status, people with HS can achieve comparable upper limb functional gains to those with IS at 3 months. Other cohort studies have also described differences in functional recovery profiles between HS and IS, with HS frequently showing greater initial disability but similar or even larger recovery over time, albeit with a slower progression (Ween et al., 1996; Kelly et al., 2003; Paolucci et al., 2016). In our cohort, this pattern was not clearly evident in the game-derived metrics, which did not mirror the functional trends reported in the clinical literature. This discrepancy is likely related to several factors, including the small HS sample, the heterogeneity of lesion characteristics and clinical severity, and the use of Armeo game scores without complementary clinical scales to characterize baseline impairment and recovery. In addition, our analyses were restricted to the first 20 rehabilitation sessions, which prevents us from characterizing longer-term recovery profiles.

Regarding PD, upper limb impairment is often dominated by bradykinesia, reduced movement speed, impaired coordination, and timing abnormalities (Capato et al., 2023). Such features are only partly reflected in the aggregate performance scores used in this study, especially for Armeo games that primarily address gross proximal control rather than fine, rhythm- and speed-dependent movements.

Our findings also indicate that not all Armeo®Spring exercises are equally informative as longitudinal outcome measures. Roll the Ball and Fly High (Elbow) yielded wide score variability and clear, disease-specific slopes, suggesting that they may be more sensitive to change in MS and IS and, to a lesser extent, in HS. Other games showed more modest or inconsistent recovery profiles. Balloons game was characterized by high baseline scores and minimal change over time, consistent with a ceiling effect or with its use as a relatively simple, low-challenge task rather than a sensitive marker of recovery. Pirate Adventure and Goalkeeper, which impose greater demands on visuomotor coordination, rapid selective responses and distal control (e.g., forearm pronation–supination), produced smaller or less consistent improvements, particularly in patients with PD. Altered motor timing, bradykinesia and potential cognitive or attentional difficulties may limit the extent to which performance on these tasks can improve over a relatively short training window. Taken together, these patterns suggest that Armeo exercises differ in their responsiveness to change and in the specific motor components they address. This task-specific responsiveness underscores the importance of carefully selecting which games to monitor as digital biomarkers of rehabilitation progress and of tailoring exercise choice to the predominant motor deficits of each neurological condition (Garro et al., 2021). For PD, for example, VR tasks explicitly designed to stress movement speed, rhythmicity and externally cued coordination might provide more informative metrics than exercises focused primarily on proximal range of motion.

In terms of practical personalization approach, our results link the responsiveness of each Armeo®Spring game to the patient’s primary motor limitations. When reduced proximal mobility and control are prominent, Roll the Ball and Fly High (Elbow), which showed the clearest longitudinal sensitivity in MS and IS, may be prioritized to target shoulder–elbow control and continuous tracking, while also serving as sensitive indicators of progress across sessions. In contrast, games that place greater demands on visuomotor coordination and distal control, such as Pirate Adventure and Goalkeeper, may be more informative when impairments in coordination, timing, selective responding, or pronation–supination are more evident (e.g., in PD). In these cases, training may benefit from initially limiting time pressure and introducing higher speed requirements only after performance accuracy has stabilized. Balloons, which showed high baseline scores and minimal change, could appear better suited as a low-challenge familiarization or warm-up task than as a longitudinal marker. Finally, because higher difficulty was consistently associated with lower scores and therapists seemed to increase task challenge progressively over time, assistance/support and difficulty should ideally be adjusted based on explicit performance criteria (e.g., reducing support once performance is stable and increasing difficulty only after consistent task execution), to maintain an appropriate challenge level. Importantly, these indications are hypothesis-generating and should be confirmed in prospective studies comparing game-derived metrics with standardized clinical outcomes. In the existing literature, Armeo®Spring has been predominantly investigated in single-diagnosis cohorts such as MS, stroke, spinal cord injury or PD, with clinical scales often complemented by a limited number of device-derived kinematic parameters (Gijbels et al., 2011; Zariffa et al., 2011; Olczak et al., 2022; Raciti et al., 2022). Only a few studies have systematically analysed Armeo-based performance metrics over repeated sessions, and these have mainly focused on learning effects in specific exercises in post-stroke patients or on pre–post changes in selected tasks (Gijbels et al., 2011; Colomer et al., 2013; Taveggia et al., 2016; Raciti et al., 2022). More recently, advanced analytical frameworks and machine learning approaches have been proposed to exploit robotic rehabilitation data (Quattrocelli et al., 2024), but these have generally been restricted to single pathologies and do not address cross-disease differences in task-specific recovery profiles. To our knowledge, no previous study has leveraged Armeo®Spring game-based scores from a heterogeneous neurological cohort to model disease-specific improvement slopes across multiple VR exercises over 20 consecutive sessions, while simultaneously accounting for covariates such as exercise difficulty, mechanical support, therapy dose and length of hospitalization. These recommendations should be considered hypothesis-generating and require prospective validation with clinical outcomes.

The strong and consistent effect of difficulty across all games confirms that higher task demands are associated with lower performance scores, supporting the construct validity of Armeo-derived metrics. At the same time, the positive correlations between difficulty, days of hospitalization, and therapy minutes observed in several disease groups suggest that therapists progressively increased task challenge over time, consistent with an adaptive training approach. This pattern was particularly evident in the MS group, where difficulty, therapy minutes, and length of stay showed stable associations across games. Notably, these trends emerged despite fatigue being a hallmark symptom of MS and a common constraint in rehabilitation. Rather than indicating that patients with MS inherently tolerate higher therapy doses, this pattern more plausibly reflects clinically guided titration of training parameters aimed at maintaining feasibility while progressively increasing challenge. Importantly, fatigue in MS is multidimensional and may involve partially distinct physical and cognitive components. In this context, mechanical support/assistance (arm/forearm unloading) is expected to primarily modulate the physical effort required for movement execution, whereas task difficulty (e.g., distractors, smaller/more numerous targets, faster scene dynamics) may preferentially increase cognitive–attentional load and executive demands. Therefore, increases in difficulty do not necessarily imply a proportional increase in physical burden and may instead reflect a gradual shift toward higher cognitive–motor challenge, while support is adjusted to keep task execution achievable. Because fatigue (and its physical vs. cognitive dimensions) was not directly assessed in this retrospective dataset, these interpretations should be considered exploratory and warrant prospective validation with standardized fatigue measures. In this sense, device logs capture not only patient learning curves but also aspects of therapists’ clinical reasoning in adjusting training intensity and complexity, even in populations for whom fatigue is a prominent concern. By contrast, age and sex did not show systematic associations with performance after adjustment for other covariates. However, given the limited sample size and the absence of detailed information on disease severity and comorbidities, these findings should be interpreted with caution.

This study has several limitations. First, its retrospective, single-center design limits causal inference and may reduce the generalizability of the findings to other settings. Second, we only included patients who completed at least 20 Armeo®Spring sessions. This threshold was chosen a priori to ensure a minimum and clinically meaningful exposure to the intervention, in line with typical upper limb robotic rehabilitation schedules. However, the time required to complete 20 sessions may vary substantially across patients and services (e.g., hospitalization logistics, economic/organizational constraints, and individual tolerance); therefore, our findings should be interpreted as session-based rather than time-based effects. This criterion may have introduced selection bias towards more adherent and clinically stable patients, thereby excluding individuals who discontinued treatment early because of medical complications, reduced tolerance or organizational constraints. Third, the outcomes were limited to device-derived game scores, without concurrent clinical scales, preventing direct inferences about changes in functional independence or activities of daily living. The relationship between these game-based metrics and standardized clinical assessments remains to be clarified. Fourth, important prognostic variables such as lesion characteristics, baseline motor severity, cognitive status and comorbidities were not systematically available, which likely contributes to the residual heterogeneity observed within each disease group and limits the ability to adjust for potential confounders. Fifth, we modeled linear trajectories over the first 20 sessions only; non-linear learning curves, early rapid gains and longer-term plateaus may not be adequately captured by this approach. Finally, the large number of correlations and statistical tests, although controlled using false discovery rate procedures, should be regarded as exploratory and hypothesis-generating rather than confirmatory.

Future prospective, multicenter studies combining robotic metrics with standardized clinical assessments and longer follow-up are needed to validate these findings and to establish the clinical utility of Armeo-derived game scores as potential digital biomarkers of upper limb rehabilitation. In addition, further research in this field could support clinicians in designing pathology-specific, tailored rehabilitation protocols and in using robotic performance recovery profiles, alongside clinical scales, to monitor patients over time during RAT.

## 5 Conclusion

Across the first 20 sessions of upper-limb robotic training with gamified, performance-based tasks, improvements were more consistently captured in task types emphasizing proximal shoulder–elbow control and continuous target tracking, whereas changes were less uniform in tasks requiring greater timing, rapid visuomotor coordination, and distal control. In our dataset, this pattern was most evident in Armeo®Spring tasks predominantly engaging proximal control (e.g., Roll the Ball and Fly High–Elbow), which may be particularly sensitive to early gains in active range and movement consistency. Baseline performance differed across diseases and exercises, and some tasks (e.g., Balloons) showed high initial scores with minimal session-to-session change, suggesting ceiling effects and limited sensitivity as short-term markers. Overall, these findings support a task-demand–driven personalization approach—selecting and progressing exercises based on the patient’s dominant motor constraints and titrating assistance/difficulty to maintain an appropriate challenge level. Although specific games are platform-dependent, this principle may generalize across robotic and VR-based rehabilitation systems. Prospective studies integrating device-derived metrics with standardized clinical scales are needed to validate these observations and inform individualized rehabilitation planning.

## Data Availability

The raw data supporting the conclusions of this article will be made available by the authors, without undue reservation.
