# Supplementary material for: Data-driven analysis of Armeo Spring performance across neurological disorders: implications for personalized upper limb neurorehabilitation
Source: Front Robot AI. 2026 Feb 13;13:1773515. doi: 10.3389/frobt.2026.1773515 (PMC12945762; doi:10.3389/frobt.2026.1773515)
Supplement: Supplementary file 1 [file Supplementaryfile1.docx]

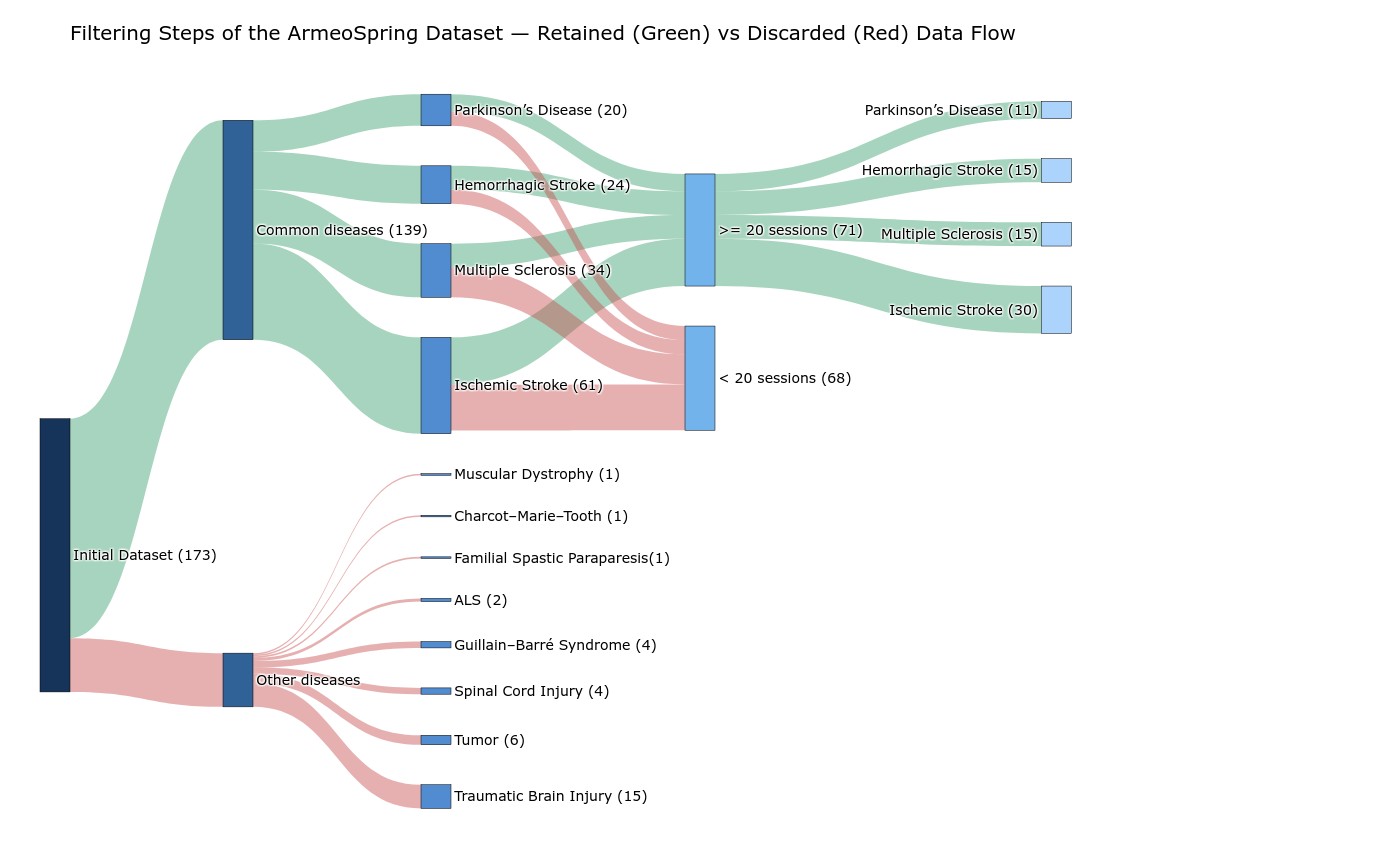


**Figure S1.** Sankey diagram representing the progressive filtering steps applied to the ArmeoSpring dataset. The initial dataset included 173 patients with various neurological conditions. After retaining only the most represented pathologies, IS, HS, MS, PD, the dataset was reduced to 139 patients who performed ArmeoSpring-based rehabilitation sessions. A subsequent filtering step excluded patients who completed fewer than 20 sessions, resulting in a final cohort of 71 patients (IS: 30; MS: 15; HS: 15; PD: 11). Green links indicate the retained data flow, whereas red links represent discarded subsets.


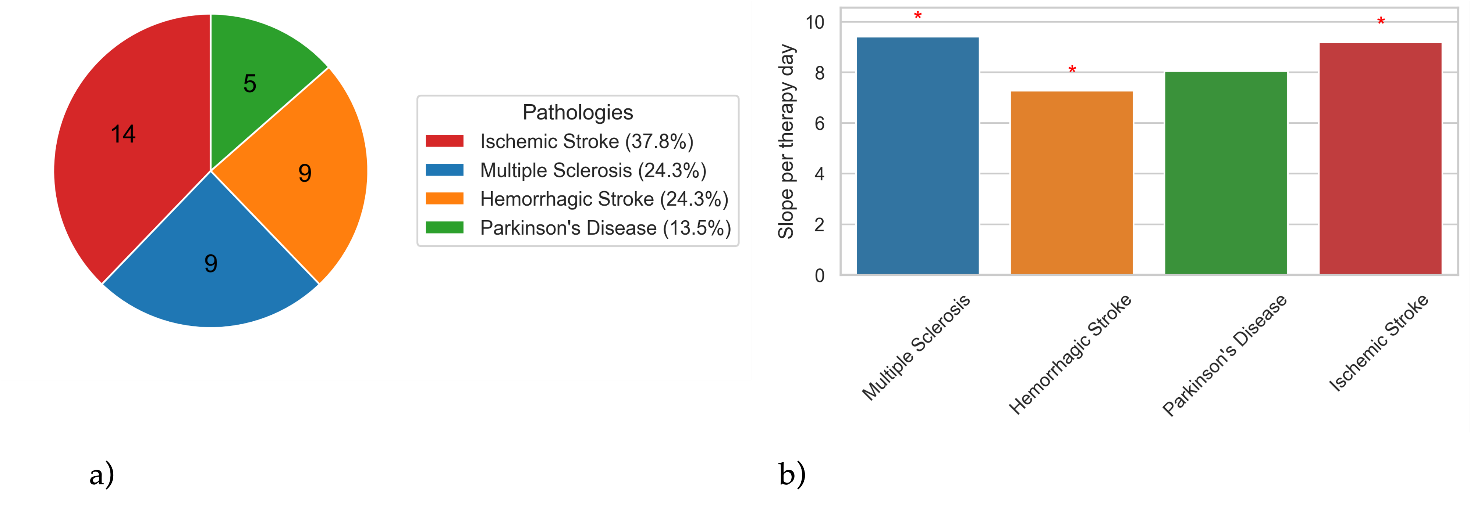


**Figure S2**. Patient composition and improvement rate for the Roll the Ball exercise. Panel a) shows the distribution of patients by neurological pathology, with absolute counts and percentages. Panel b) presents the mean slope of improvement in game performance per therapy day, separated by pathology. Red asterisks on top of bars mark statistically significant improvements after FDR correction (FDR < 0.05) for Multiple Sclerosis, Hemorrhagic Stroke and Ischemic stroke patients.


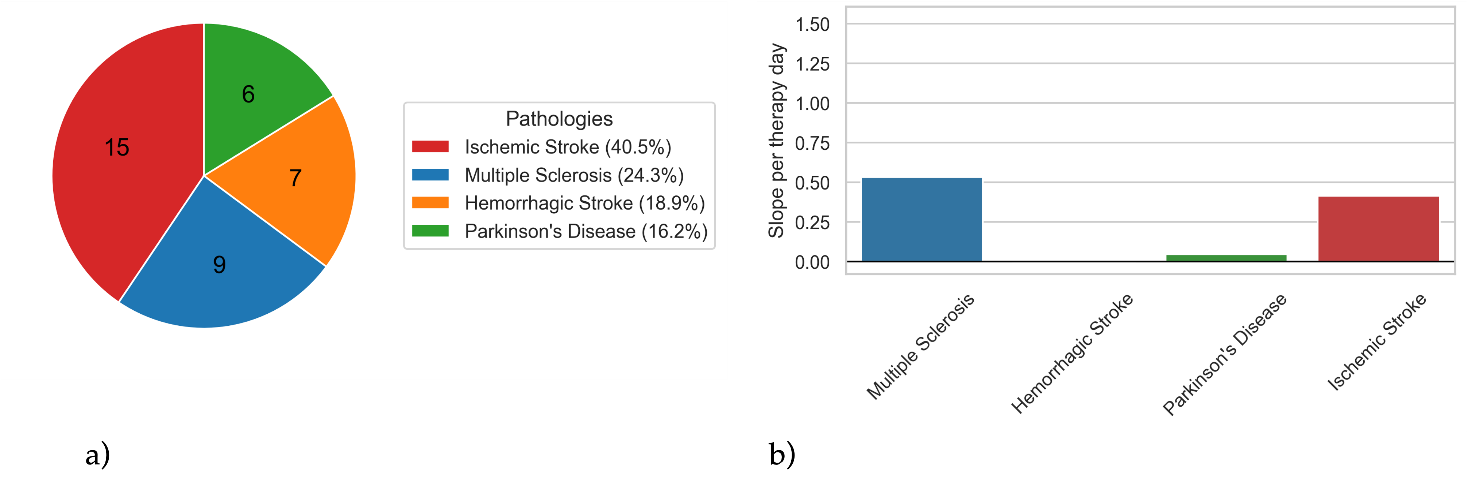


**Figure S3**. Patient composition and improvement rate for the Goalkeeper exercise. Panel a) shows the distribution of patients by neurological pathology, with absolute counts and percentages. Panel b) presents the mean slope of improvement in game performance per therapy day, separated by pathology. No statistically significant improvements after FDR correction (FDR < 0.05) were identified for this exercise.


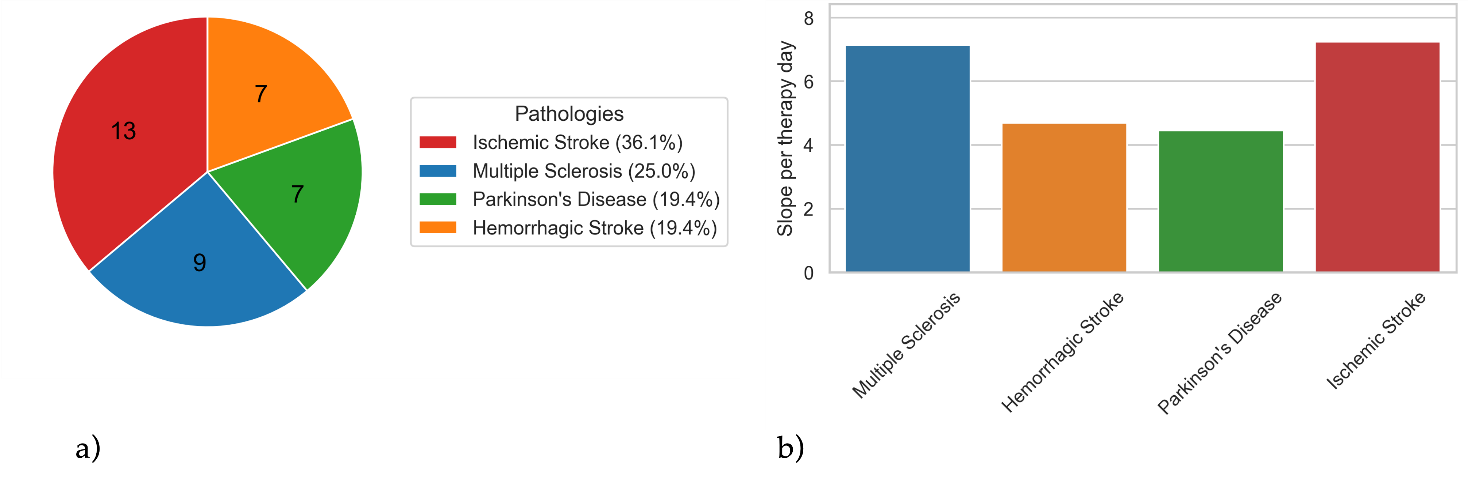


**Figure S4.** Patient composition and improvement rate for the Balloons exercise. Panel a) shows the distribution of patients by neurological pathology, with absolute counts and percentages. Panel b) presents the mean slope of improvement in game performance per therapy day, separated by pathology. No statistically significant improvements after FDR correction (FDR < 0.05) were identified for this exercise.


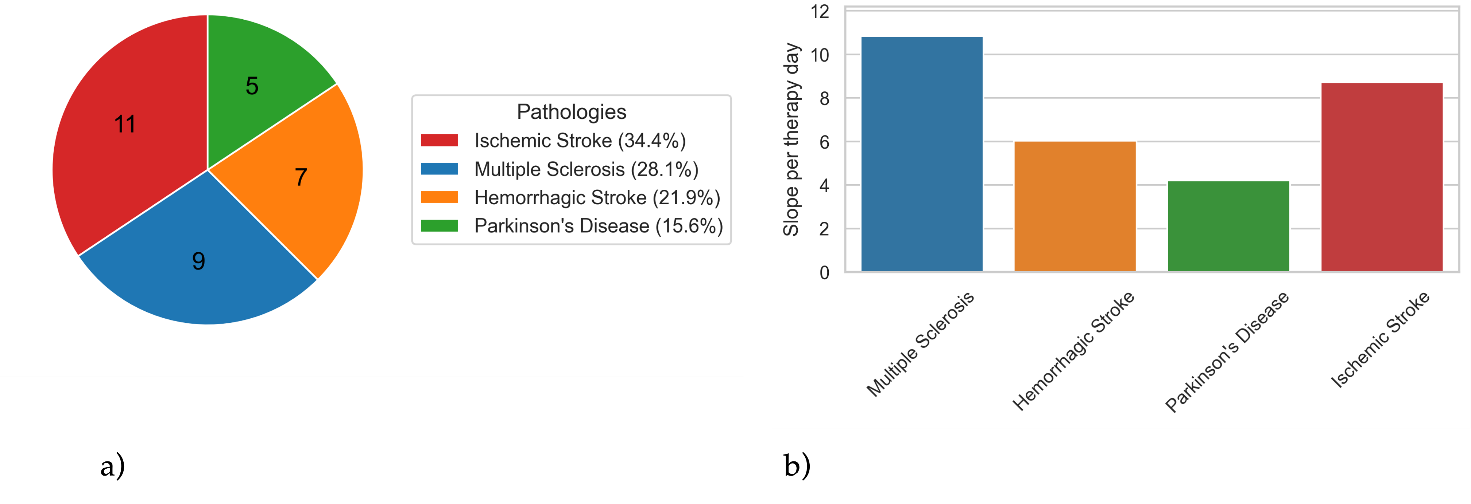


**Figure S5.** Patient composition and improvement rate for the Pirate Adventure exercise. Panel a) shows the distribution of patients by neurological pathology, with absolute counts and percentages. Panel b) presents the mean slope of improvement in game performance per therapy day, separated by pathology. No statistically significant improvements after FDR correction (FDR < 0.05) were identified for this exercise.


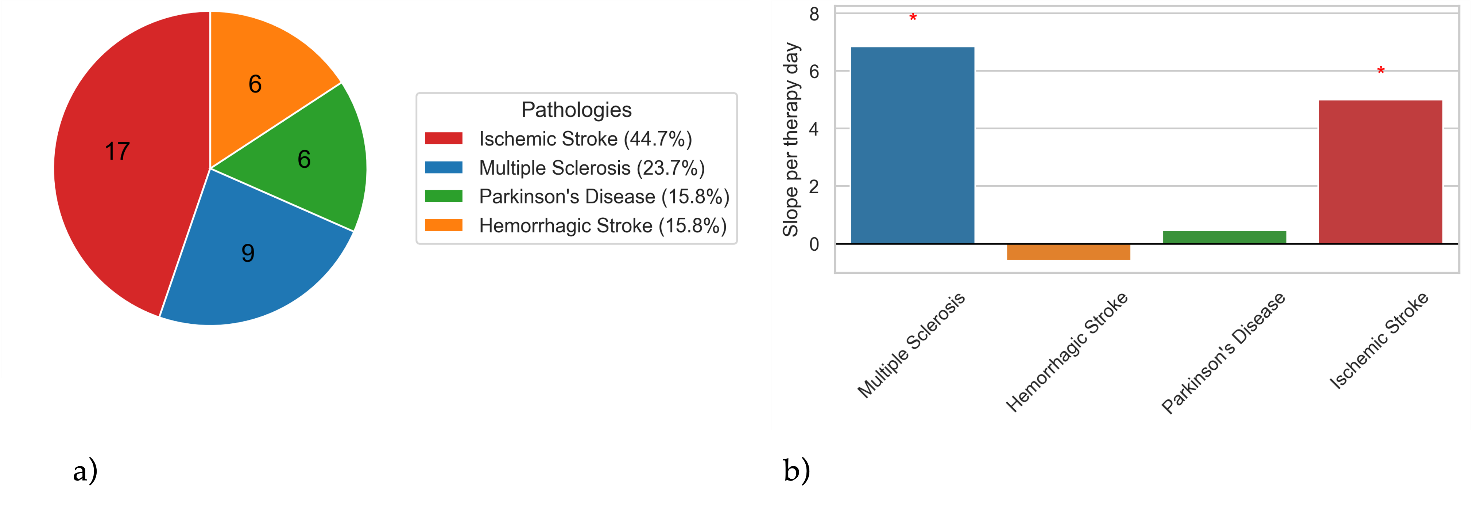


**Figure S6.** Patient composition and improvement rate for the Fly High Elbow exercise. Panel a) shows the distribution of patients by neurological pathology, with absolute counts and percentages. Panel b) presents the mean slope of improvement in game performance per therapy day, separated by pathology. Red asterisks on top of bars mark statistically significant improvements after FDR correction (FDR < 0.05) for Multiple Sclerosis, and Ischemic stroke patients.
